# Toxic Metals (Pb and Cd) and Their Respective Antagonists (Ca and Zn) in Infant Formulas and Milk Marketed in Brasilia, Brazil

**DOI:** 10.3390/ijerph7114062

**Published:** 2010-11-18

**Authors:** Clarissa S. P. De Castro, Andréa F. Arruda, Leandro R. Da Cunha, Jurandir R. SouzaDe, Jez W. B. Braga, José G. Dórea

**Affiliations:** 1 Laboratório de Tecnologias para a Segurança Alimentar, Embrapa Recursos Genéticos e Biotecnologia, P.O. Box 02372, 70.770-917, Brasília-DF, Brazil; E-Mails: clarissa@cenargen.embrapa.br (C.S.P.C.); lelenut@yahoo.com.br (L.R.C.); 2 Laboratório de Eletro e Espectroanalítica, Instituto de Química, Universidade Federal de Goiás, P.O. Box 131, 74.001-970, Goiânia-GO, Brazil; E-Mail: arruda@quimica.ufg.br; 3 Laboratório de Química Analítica e Ambiental, Instituto de Química, Universidade de Brasília, P.O. Box 04394, 70.919-970, Brasília-DF, Brazil; E-Mails: rodsouza@unb.br (J.R.S.); jez@unb.br (J.W.B.B.); 4 Laboratório de Bioquímica da Nutrição, Faculdade de Saúde, Departamento de Nutrição, Universidade de Brasília, P.O. Box 04322, 70.919-970, Brasília-DF, Brazil

**Keywords:** cadmium, calcium, formulas, infant nutrition, lead, milk, zinc

## Abstract

In non-ideal scenarios involving partial or non-breastfeeding, cow’s milk-based dairy products are mainstream in infant feeding. Therefore, it is important to study the concentrations of potentially neurotoxic contaminants (Pb and Cd) and their respective counteracting elements (Ca and Zn) in infant dairy products. Fifty-five brands of infant formulas and milk sold in Brasilia, Brazil were analyzed. The dairy products came from areas in the central-west (26%), southeast (29%) and south of Brazil (36%) extending as far as Argentina (7%) and the Netherlands (2%). For toxic Pb and Cd, median concentrations in powdered samples were 0.109 mg/kg and 0.033 mg/kg, respectively; in fluid samples median Pb concentration was 0.084 mg/kg, but median Cd concentration was below the limit of detection and overall values were below reference safety levels. However, 62% of these samples presented higher Pb concentration values than those established by FAO/WHO. Although the inverse correlation between Cd and Zn (Spearman r = −0.116; P = 0.590) was not statistically significant, the positive correlation between Ca and Pb was (Spearman r = 0.619; P < 0.0001). Additionally, there was a significant correlation between Pb and Cd. Furthermore, the study also revealed that provision of the essential trace element Zn in infant formulas can provide adequate amounts of the recommended daily requirements. Infant formulas and milk sold for consumption by infants and children can be an efficient tool to monitor neurotoxic metal risk exposure among young children.

## 1. Introduction

Breastfeeding is necessary for optimal growth and development of infants. There are innumerable and unique health and neurodevelopment advantages to short and long-term breastfeeding. Nevertheless partial and non-breastfeeding take place worldwide. The occurrence of necessary essential nutrients and micronutrients in cow’s milk has made it the alternative food of choice for the human infant. In such non-ideal circumstances infant feeding practices have relied on technical advances in the food industry to improve upon mainstream cow milk-based products. In this realistic scenario, it should be recognized that pasture and animal foodstuffs [1] can be contaminated with pollutants and, as a consequence, increase the risk of the non-breastfed infant being exposed to hazardous substances.

Grazing land can be contaminated with Pb and Cd from parent materials, atmospheric deposits, continuous application of large amounts of fertilizer, disposal of industrial waste sludge and vehicle emissions [2]. In addition to that, packaging and technological processes used to bring foods to the consumer can significantly increase the total concentration of Pb and Cd [3,4]. Therefore, potentially toxic elements (Cd, Pb) deriving from environmental pollution or accidental contamination may be transferred to cow’s milk and consumed by vulnerable infants.

Intake of contaminated milk containing low levels of Cd and Pb can cause various clinical abnormalities or may pass without clinical signs [5]. However, studies have shown that dietary intake of selected nutritional elements might help counteract the effects of cadmium and lead. Animal studies showed that the amount of calcium in food is related to tissue lead accumulation [6–9]. Indeed, epidemiological studies have shown that food calcium content is inversely correlated with lead in blood, bone or hair [10–12]. Recently, rates of absorption and whole-body retention of dietary Cd were increased 7 to 10-fold when experimental animals were fed diets containing marginal concentrations of Zn, Fe, and/or Ca [13,14]. Therefore, our objective was to assess the occurrence of toxic cadmium and lead along with the natural nutritional elements (zinc and calcium) in cow’s milk and infant formulas sold in Brasília, Brazil.

## 2. Experimental

### 2.1. Apparatus

Microwave digestion of milk samples was performed on a DGT-100 (Provecto Sistemas, Brazil) microwave system. The measurement of Zn, Cd and Pb in milk samples by anodic stripping voltammetry (ASV) were carried out in a 747 VA Computrace instrument (Metrohm, Switzerland) equipped with an electrochemical cell composed of a hanging mercury electrode (working electrode), Ag/AgCl (3.0 mol L^−1^ KCl) electrode (reference electrode) and a platinum electrode (auxiliary electrode). As a supporting electrolyte 0.2 mol L^−1^ sodium acetate was used. All ASV measurements were performed in the potential range of −1.15 V (initial potential, E_i_) to 0.20 V (final potential, E_f_) at the following settings: accumulation potential E_d_ = −1.15 V, accumulation time t_d_ = 180 s, equilibration time t_e_ = 20 s, surface area of the mercury drop 4 mm^2^, pulse amplitude 59.5 mV and scan rate v = 10 mVs^−1^. An AA 400 Flame Atomic Absorption Spectrometer (Perkin Elmer, USA) with calcium hollow cathode lamp (422.7 nm) was used for calcium determination in milk samples. The optimum operation conditions were 0.2 nm band width, 20 mA lamp current and 2.5 mL min^−1^ acetylene flow rate in an air/acetylene flame. The pH of the solutions was determined using a 3030 Jenway pH-meter (United Kingdom) with a DME-CV1/Digimed combination pH electrode (Brazil).

### 2.2. Chemicals and Samples

Analytical-reagent grade chemicals and ultrapure water (Millipore, USA) were used to prepare all solutions. Nitric acid, hydrogen peroxide, sodium acetate and cadmium, lead and zinc stock standard 1,000 mg L^−1^ solutions were purchased from Merck (Darmstadt, Germany). Calcium stock 1,000 mg L^−1^ solutions were prepared using CaCl_2_·5H_2_O (Aldrich, USA) in HNO_3_ 5% (Merck, USA). We searched major supermarkets for dairy products used in infant feeding, *i.e.*, milk and infant formulas. All information regarding processing plants was obtained from product labels. Fifty-five milk samples, which corresponded to the 22 most widely accepted and consumed brand names in the Federal District, were purchased from local supermarkets in Brasília, Brazil, in March 2007.

### 2.3. Procedure

The laboratory glassware was kept in a 5 or 20% (v/v) nitric acid solution overnight. Afterwards, it was rinsed thoroughly with ultrapure water and air-dried. Milk samples were prepared to carry out the voltammetric and spectroscopic analyses using the optimized procedure as described.

An aliquot of each milk sample (0.4 g for powered samples and 1.0 mL for liquid samples) was weighed into a Teflon digestion vessel. Afterwards, 65% nitric acid (5.0 mL) and 30% hydrogen peroxide (2.5 mL) were added and samples subjected to closed vessel microwave digestion. The optimized microwave digestion program was performed at 350 W (8 min), 550 W (8 min), 700 W (7 min), 0 W (6 min) and 850 W (5 min). At the end of the digestion process, the digests were diluted to 25 mL with double-distilled water quality for the measurement of Zn, Ca, Pb and Cd.

The measurement of Zn, Cd an Pb in milk samples was accomplished through multiple standard additions of 100 μL of 1.00 × 10^−4^ mol L^−1^ Zn^2+^, 300 μL of 1.00 × 10^−5^ mol L^−1^ Cd^2+^ and 300 μL of 1.00 × 10^−5^ mol L^−1^ Pb^2+^ to the electrochemical cell containing 20 mL of 0.2 mol L^−1^ sodium acetate and 1.0 mL of pretreated milk sample. To avoid measurement interference due to metal adsorption on the working surfaces of the electrode system, electrodes were submitted to periodic cleaning with 20% HNO_3_ (by volume) followed by a generous wash with ultrapure water. Experiments were performed in three replicates at 23 °C and pH 4.0, and preceded by a gentle N_2_ bubbling to prevent oxygen diffusion into the electrochemical cell (10 min for the supporting electrolyte and 1 min after metal addition).

The determination of calcium levels was carried out using multiple standard additions in milk samples. Five solutions containing 1.0 mL of pretreated milk sample in each were prepared by additions of 0, 50, 100, 150 and 200 μL of 1.00 × 10^−3^ mol L^−1^ Ca^2+^ for a 50 mL final volume. In all samples the measurements were done in three replicates.

Nine parameters have been evaluated for the validation of analytical methods in milk, namely: selectivity, repeatability, reproducibility, linearity range, detection and quantification limits, recovery, stability and control charts. The limits of detection and quantification, and linearity range of Zn, Cd, Ca and Pb were determined using analytical curves constructed by multiple standard additions. The analyte stability was characterized by analyzing a pretreated infant formula (L5) sample once a week during eight weeks. The trueness of the analytical methods was assessed by recovery assays. Known amounts of Zn, Cd, Pd and Ca were added to three infant formulas (L1 = 5.700 mg/g Ca; 0.0627 mg/g Zn; 0.4000 mg/kg Cd; 0.0037 mg/g Pb; L2 = 4.300 mg/g Ca; 0.0052 mg/g Zn; 0.400 mg/kg Cd; 0.0027 mg/g Pb; L3=5.700 mg/g Ca; 0.0051 mg/g Zn; 0.4 mg/kg Cd; 0.0016 mg/g Pb). The repeatability was determined by analyzing a pretreated infant formula (L5) sample five times in the same day. The reproducibility was determined by analyzing a pretreated infant formula (L5) sample eight times in different days (one time per week). The selectivity was evaluated by comparing the slopes of the analytical curves obtained with aqueous standards and with standard additions to a pretreated infant formula (L5) sample. The control charts (R *vs*. time) were constructed based on stability data, where R is the difference between the lower and higher replicate values in each week.

### 2.4. Statistical Analysis

Data were summarized with Microsoft Office EXCEL software (version 2007; Microsoft Corp, Redmond, WA, USA). Non-parametric Spearmann correlation tests between variables were done with PRISM software (version 4.0; San Diego, CA, USA); the significance level was set at P < 0.05. We applied Hierarchical Cluster Analysis (HCA) to identify patterns of dairy farms with comparable concentrations of determined elements. After identification of distribution patterns we applied Principal Component Analysis (PCA); concentrations below detection limit were attributed a zero value.

## 3. Results

The overall performance of the proposed methods is summarized in Table 1. The limits of detection (LOD) and limits of quantification (LOQ) were calculated from the expressions LOD = 3S_b_/b and LOQ = 10S_b_/b, where S_b_ is the standard deviation of the blank and b is the slope of the analytical curve [15]. The results for the recovery tests were within the acceptance range 90–110%, especially considering the thermal microwave digestion program used for milk samples. The stability results showed recoveries (obtained during the five weeks) in good agreement with the recovery of the method, except for Cd. It was observed that Cd recovery had a slight decrease in the second week under the used storage conditions; random fluctuations were observed. Selectivity studies were performed in order to investigate the effect of potential interference of the matrix. For Cd, Pb and Ca, the slopes obtained with aqueous standards were similar (95% confidence level) to those obtained for the matrix standard additions. Therefore, there was no matrix interference in the tested concentration range. The repeatability and reproducibility were calculated after Waeny [16]. The method proposed by Waeny [16] takes into consideration the standard deviation for each set of measurements over a spread index (95% confidence level) for a pretreated sample analyzed *n* times (degree of freedom = n − 1). The calculated repeatability and reproducibility values were considered satisfactory; repeatability index was smaller than reproducibility index for all four elements. Indicating that the between days measurements variation were bigger than the same day variation. A control chart was constructed for each element data during eight weeks and was applied to monitor the analytical process variation and efficiency. Based on upper control limits (UCL=D4 *R̄*) and lower control limits (LCL=D3 *R̄*); *R̄*is the average of the R values obtained during each week, and D is the limit constants [17]. The estimated R values were within limits. Therefore, the process performance displayed consistency.

The concentrations of metals in samples of milk and formulas as a function of brands are shown in Tables 2 and 3. Table 2 details powdered products while Table 3 illustrates fluid milk. In all (55), these fluid and powder milk samples represent products from animals raised on farms from the southeast (16 samples: two from Rio de Janeiro, three from São Paulo and 11 from Minas Gerais), south (20 samples: three from Paraná, 13 from Rio Grande do Sul and four from Santa Catarina) and central-west (14 samples: 14 from Goiás) states of Brazil. Some samples were also produced in Argentina (four samples) and in The Netherlands (one sample). As regards Cd, the median concentration (below detection limit in fluid samples) is lower than the reference value recommended by the Brazilian Government (1.0 mg/kg) [18]. However, the median concentration of Pb (0.109 mg/kg) is higher than the maximum limit established by the FAO/WHO (0.02 mg/kg) [19]. Therefore, in seven infant formulas and in 22 fluid samples from different regions of Brazil, Pb concentration values were higher than recommended by the Brazilian Government (0.2 mg/kg for infant formulas and 0.05 mg/kg for fluid milk) [18].

The low Cd concentrations do not seem a potential exposure risk. However, it is worth mentioning that Zn (as an essential nutrient) when consumed in these products it does not show the physiological variation found in breastfeeding. Provided at uniform concentrations in cow’s milk preparations, it may not be ideal as the sole source of infant nourishment [20]. Indeed, despite its nutritional importance most of the products sampled (75%) did not mention Zn concentrations on packaging. Among the infant formulas analyzed in this study in which concentrations of zinc were available (14%), only 28% showed values with an error of less than 10% when compared with the values printed on packaging.

Nevertheless, ten of the powdered samples (six from the southeast of Brazil, one from the south of Brazil, two from Argentina and the one from The Netherlands) presented Zn concentration values above the maximum limit established by the Brazilian Government (50 mg/kg) [18]. Calcium contents showed the same profile, with most of the samples differing from the values listed on the labels: powdered samples were 20% statistically similar, 35% below and 37% above the label values. Twenty-eight percent of liquid samples showed values within a 10% error range of the declared values and 23% presented concentrations two to three times lower than declared on packaging.

**Figure 1:**
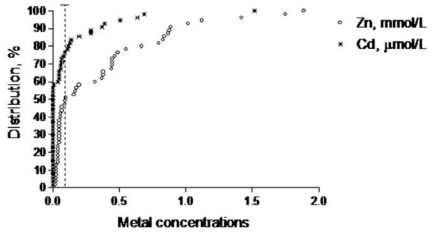

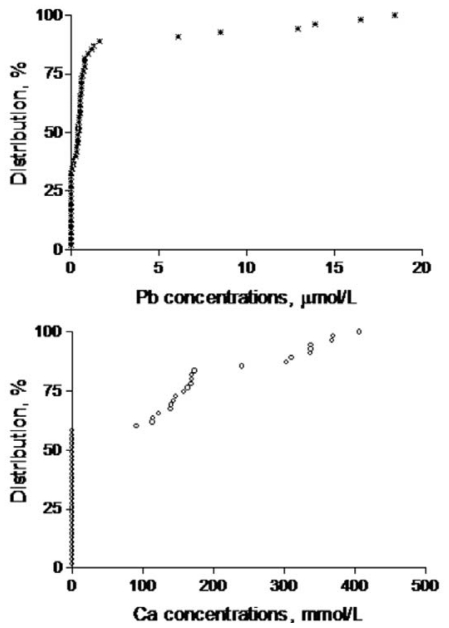
Frequency distribution of toxic (Pb and Cd) and essential elements (Ca and Zn) in cow’s milk consumed by children in Brasília; values below the detection limit are represented as zero.

**Figure 2:**
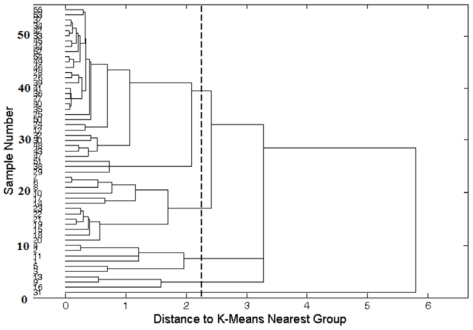
Dendrogram obtained by analysis of the data matrix containing 55 samples and four variables (metals: Zn, Pb, Cd and Cu).

Data representations on a molar basis as frequency of distributions are illustrated in Figure 1 for the determined elements. The correlation between Cd and Zn (Spearman r = −0.116; P = 0.590) was not statistically significant, but the correlation between Ca and Pb (Spearman r = 0.619; P < 0.0001) was. However, there was a significant correlation between Pb and Cd (Spearman r = −0.532; P = 0.016) within milk samples where both metals were measured above limit of detection. HCA was applied to identify patterns of the determined element concentrations and geographical origin of the milk samples, which are shown as a dendrogram in Figure 2; there are five distinct clusters. It was observed that L31 appeared in an isolated group, indicating that this sample presents distinct characteristics; however, this sample is originated from the same region as L30, L50, and L54. The PCA was applied in order to reduce the number of variables and to improve visualization of the data. Table 4 presents the explained variance of each principal component (PC). Almost 71 and 90 % of the total variance are explained by the first two and three PC, respectively.

**Figure 3:**
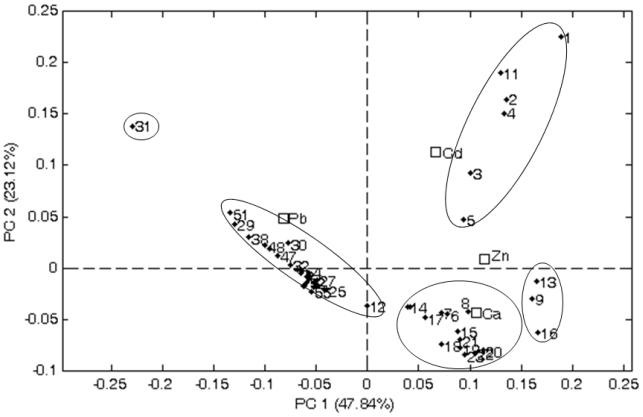
Two-dimensional representation of the spatially distribution of scores and weights determined by first two principal components (PC) of PCA.

Figure 3 shows the biplot graph of the scores and weights; the suggested classes of hierarchical clustering analysis are represented by ellipses. It can be observed that samples L1, L2, L3, L4, L5 and L11 are grouped in a quadrant with positive values in PC1 and PC2. For these samples the concentration of cadmium is responsible for the shown behavior; this metal is the variable that contributes for positive values in both PC1 and PC2. It is interesting to notice that these samples came from the same dairy region of Ibiá - Minas Gerais (MG). Indeed, results shown in Figure 3 and Table 2 show that the high value in PC1 is approximately inversely related to the concentration of cadmium; furthermore, all samples with positive values in PC1, except for sample L3, present undetectable concentrations of cadmium.

In the quadrant with positive values of PC1 and negatives of PC2 it can be noticed that samples L9, L13 and L16 show a small separation from a larger group of samples, and that both groups are distinguished from others mainly by different concentrations of Ca and Zn. Considering all samples present in this quadrant, four distinct dairy regions are observable: Buenos Aires-Argentina (4), Ibiá, Ituiutaba and Sete Lagoas, Minas Gerais (4), Araçatuba-SP (2), Teutônia-RS (2), Cordilheira Alta-SC (1), Bela Vista de Goiás-GO (1) and Itaperuna-RJ (1). It is noteworthy that the localities of Minas Gerais can be considered as the same dairy region; they are equidistant from Ibia, Ituiutaba and Sete Lagoas, which correspond to 23.5% of the samples. Teotônia-RS and Cordilheira Alta-SC can also be considered as belonging to the same dairy region despite being 370 km apart (18% of the sample set).

A third group containing a substantial number of samples is observed in the negative region of PC1 and extending from negative to positive values of PC2. Since Pb is the element next of this group and located approximately at the diagonal of the PC1-PC2 plot, its concentration is the most important for the separation of this group. Finally, it is observed that the sample L31 is distant from all other samples, suggesting that this sample presents distinct characteristics from all samples in the data set, which is in agreement with the results presented in the dendogram. It is important to notice that if three PC were considered for this pattern recognition any differences are observed in the clusters identified. From the 31 samples that make up this group, 14 (45%) comes from the cities Concórdia-SC, Carazinho, and Arroio do Meio-RS, where Carazinho is equidistant from these two cities, about 180 km from each. The cities Bela Vista de Goiás (6), Rianápolis (1), Sao Luís de Montes Belos (1), Morrinhos (2), Corumbaíba (1), Santa Helena de Goiás (1) and Hidrolândia (1) are the source 42% (13 out of 31) of the samples. These cities are located within a radius of 200 km from the Goiânia - GO and can be considered as a single dairy region. The region of Carambeí-PR is the origin of 9.7% (three out of 31) samples and the region of Amparo-SP is the origin of just one sample.

## 4. Discussion

The main contribution of this study is to demonstrate the feasibility of a pragmatic approach to using commercially available infant formula (prepared from cow milk) and commercially produced milk as a tool to monitor risk of early exposure to environmentally hazardous elements. In this study we found that dairy animal feeding practices in Brazil and in Argentina operate in an environment conducive to maintaining Cd levels in milk products below optimum thresholds. It was also revealed that although the correlation between Cd and Pb was statistically significant, their occurrence had a distinct spatial pattern. While elements such as calcium and zinc in infant formulas are not a nutritional issue *per se*, their occurrence as antagonists to toxic Cd and Pb merits consideration. Furthermore, of particular public health interest is that Pb concentrations above threshold limits have been confirmed in the studied samples. Most of the infant formulas showed Zn concentrations above limits set by Brazilian regulators. Because Zn transfer from blood to milk is tightly controlled (at least in breast milk [20]), these high values may result from milk processing methods.

Our results indicate that we do not currently have potential sources of contamination in areas of dairy farming for Cd. However, it is of concern that 62% of the sampled products showed Pb concentrations above reference values provided by the FAO/WHO [19]. This should be of concern because of the potential risk posed to formula-fed infants (especially more vulnerable preterms and newborns). Improper dilution of powdered milk products is frequently found, leading to overconcentration during reconstitution [21] which can potentially increase the exposure to toxic contaminants. It should be emphasized that nevertheless the ultimate exposure of nursing infants fed cow’s milk or formulas will depend on the concentrations of toxic elements in the running water used during reconstitution of the powdered product [1].

Nutritional as well as toxic element concentrations in cow’s milk vary widely due to constitutional (animal breed and lactation period) and environmental conditions related to geographic factors (climate, season, and soil contamination) and especially dietary composition of animal feed and manufacturing practices of final products. The concentrations of Cd and Pb that we found are above values reported by others in Brazil. In milk sold in Rio de Janeiro city, concentrations of Pb and Cd were, respectively, 140 μg/kg and 1.1 μg/kg [22]. In fresh and pasteurized bovine milk from the Paraíba Valley region, the mean concentrations of Pb have been reported as 0.04 and 0.23 mg/L, while Cd was below detection limits [23,24]. In the Northern state of Pernambuco the concentration of Pb in fresh milk samples varied from 3 to 90 μg/L [25].

These results are in agreement with reports from other regions of Brazil [22–25]. Indeed, it has been suggested that the largest contribution of lead contamination in cow’s milk (74%) in Brazil arises from the metal vessels used to transport milk to the processing plants [26]. At least that was the explanation offered for high concentrations of lead (0.25 mg/L) reported in cow-milk samples collected from a highly industrialized area (Paraiba do Sul river valley) in Brazil [26]. Indeed, contamination of containers is often reported as a cause for concern in Pb and Cd contamination of dairy products [27,28]. It seems that this trend is observed in other parts of the world; Kazi *et al.* [29] reported that in Pakistan, milk Pb and Cd concentrations were higher in processed milk than in raw milk at the farms.

In regions of Italy with very low industrial activity or automotive pollution (Calabria), cow-milk mean concentrations of Zn (2,016 μg/kg), and Cd (0.02 μg/kg) were comparable to those in our work but mean Pb (1.32 μg/kg) was much below our median value [30]. Also in Italy, infant formula concentrations of Cd and Pb varied from 1.0 to 3.7 μg/kg and from 4.9 to 19.2 μg/kg [31]. Mean concentrations of cadmium and lead in milk samples purchased from Mumbai (India) were, respectively, 1.6 and 0.1 μg/kg [32].

Concentrations of Pb (1.47 ng/mL) and Cd (25 ng/mL) in cow’s milk from Argentina are higher than median values reported in our work [33]. The concentrations of Cd and Pb in milk and milk-dairy products collected in the local supermarkets of Izmir (Turkey) varied from 0.08 to 674.3 μg/L and from 0.15 to 2.94 mg/L, respectively [34]. The mean Cd and Pb contents obtained from 36 milk samples collected from the bulk holding tanks of dairy farms in 12 localities in Izmit (Turkey) were 0.257 (0.180–0.398 μg/L) μg/L and 6.83 (5.32–9.94 μg/L) μg/L, respectively [35]. In Slovenia, cow’s milk samples from 19 dairy locations showed levels of Pb (0.07 mg/kg) and Cd (0.07 mg/kg) within the maximum residue limits [36].

Part of the variation in metal concentrations in our work may be attributed to differences in proximate composition of the dairy products. The work of Martino *et al.* [37] showed striking differences in the measured elements as a function of the analyzed fraction. Whole milk showed the highest concentrations, while the whey fraction showed the lowest concentrations. This indicates that the bulk of the elements (especially Pb and Cd) are attached to milk proteins; this may explain the significant correlation between Pb and Cd (measure above detection limits). Considering that, it is interesting to notice that in our work Cd were also lower in the infant formulas but Pb content did not show a pattern. Cow’s milk has a much higher protein concentration than human milk [38]. Therefore, most infant formulas are “humanized” to mimic human milk concentrations of nutrient (especially protein) concentrations. Protein concentrations are then reduced to circa of 1/3 of their original concentrations. As a result, most of the Cd concentrations are also proportionally diminished.

Ikem *et al.* [39] compared the cow-milk-based infant formulas from the USA, the UK and Nigeria; there was no striking difference among the countries for concentrations of Zn, Ca, Pb and Cd. Compared to Ikem’s *et al.* results [39], our study showed striking differences only for Pb concentrations. In our study some Pb concentrations were one order of magnitude higher than in the countries studied by Ikem *et al.* [39]. However, the most recent study in Brazil indicated Pb contamination of cow’s milk in a polluted region of São Paulo State (mean concentrations were higher, 0.23 mg/L), but Cd was not detected [24].

Transference of Pb and Cd along the terrestrial food chain was studied by Baranowska *et al.* [40]. Not only was concentration for Pb in soil higher than Cd, but they also showed that Pb transferred proportionally more from soil to grass and from grass to milk than Cd. It should also be noted that Zn concentrations in soil, grass and milk were higher and proportionately better transferred than Cd. It is widely recognized that prolonged bottle-feeding is associated with iron deficiency [41]. In this regard, monitoring toxic metal exposure in infancy through formulas is of great importance; Satarug *et al.* [42] have hypothesized that Cd accumulation can result from the efficient absorption and transport of cadmium, employing multiple cellular uptake used for essential elements such as calcium, iron, zinc and manganese.

## 5. Conclusions

Commercialized infant formulas and milk consumed by children can be an efficient tool to monitor environmental contamination and risk of children’s exposure to neurotoxic metal (Cd and Pb). Health regulators and manufacturers of milk formulas can take advantage of these findings to abate Pb exposure in vulnerable infants.

## Figures and Tables

**Table 1:** Validation parameters.

Metals	[Zn^2+^]/μg/L	[Pb^2+^]/μg/L	[Cd^2+^]/μg/L	[Ca^2+^]/mg/L

**Method LOD and LOQ**	1.89; 6.32	2.71; 9.04	0.62; 2.08	0.206; 0.687
**Linearity range**	31 a 244	5.3 a 42	9.8 a 77	0.69 a 208

**Performance Parameters**				

**Recovery**	92–93%	91–111%	95–100%	90–110%
**Precision**	2–5%	3–9%	2–3%	1–2%
**Accuracy**	0.001–0.19	0.9–1.6	0.02–0.63	0.01–0.08

**Table 2:** Metal concentrations in powdered samples.

Milk sample	([Zn^2+^] ± s )^a^ mg/g	([Pb^2+^] ± s ) a mg/kg	([Cd^2+^] ±s ) a mg/kg	([Ca^2+^] ± s) a mg/g
Ninho +1 (L1) b	0.0647 ± 0.0035	3.7084 ± 0.5819	---------	5.719 ± 0.128
Nestogeno2 (L2) b	0.0535 ± 0.0019	2.7971 ± 1.0983	---------	4.430 ± 0.094
Ninho +3 (L3)	0.0528 ± 0.0028	1.7148 ± 0.7589	0.0103±0.0000	5.459 ± 0.241
Nestogeno 1 (L4) b	0.0559 ± 0.0011	2.5986 ± 1.2346	---------	4.461 ± 0.296
NAN A.R (L5) b	0.0505 ± 0.0095	1.2271 ± 0.0327	---------	5.432 ± 0.333
NAN sem lactose(L6) b	0.0568 ± 0.0005	---------	---------	6.750 ± 0.303
NAN 2 (L7) b	0.0550 ± 0.0030	---------	---------	6.378 ± 0.339
Bebelac 1 (L8) b	0.0712 ± 0.0120	---------	---------	6.605 ± 0.371
Aptamil 1 (L9) b	0.1200 ± 0.0079	---------	---------	5.597 ± 0.224
Bebelac 2 (L10) b	0.0424 ± 0.0020	---------	---------	4.792 ± 0.279
Nestogeno Plus (L11) b	0.0298 ± 0.0060	3.3199 ± 1.5270	---------	6.147 ± 0.440
Aptamil 2 (L12) b	0.0205 ± 0.0023	---------	---------	3.573 ± 0.200
Elegê kids (L13) b	0.1111 ± 0.0206	0.3251 ± 0.2041	---------	6.605 ± 0.301
Ninho (L14) b	0.0285 ± 0.0029	0.1586 ± 0.0704	---------	6.584 ± 0.460
Glória integral (L15)	0.0241 ± 0.0020	0.2559 ± 0.1492	---------	13.150 ± 0.182
Itambé Desnatado (L16)	0.0901 ± 0.0109	---------	---------	11.785 ± 0.027
Ninho +6 (L17) b	0.0237 ± 0.0006	0.1964 ± 0.0000	---------	9.370 ± 0.337
Molico desnatado (L18)	0.0242 ± 0.0014	---------	---------	12.078 ± 0.352
Glória desnatado (L19)	0.0310 ± 0.0073	---------	---------	13.107 ± 0.470
Itambé integral (L20)	0.0286 ± 0.0003	0.1516 ± 0.0670	---------	15.825 ± 0.395
Elegê integral (L21)	0.0284 ± 0.0061	0.1235 ± 0.0858	---------	13.146 ± 0.222
Leo (L22)	0.0348 ± 0.0039	---------	---------	14.322 ± 0.529
Carrefour (L23)	0.0276 ± 0.0029	---------	---------	14.377 ± 0.796

a Mean values from three independent determinations.

b Infant formulas.

**Table 3:** Metal concentrations in fluid milk samples.

Milk sample	([Zn^2+^] ± s ) a mg/L	([Pb^2+^] ± s ) a mg/L	([Cd^2+^] ± s ) a mg/L	([Ca^2+^] ± s ) a g/L
Parmalat integral (L24)	11.6525 ± 1.2498	---------	---------	3.617 ± 0.236
Parmalat calcio plus (L25)	13.0846 ± 0.4000	---------	---------	1.681 ± 0.100
Parmalat Premium int. (L26)	3.3773 ± 0.2244	0.1375 ± 0.0090	---------	2.577 ± 0.182
Parmalat Premium desn. (L27)	3.3624 ± 0.5214	0.1593 ± 0.0065	---------	1.365 ± 0.070
Parmalat extra Premium (L28)	4.4896 ± 0.6655	0.0618 ± 0.0208	---------	2.423 ± 0.178
Dietlat cálcio plus (L29)	3.1960 ± 0.0046	0.0112 ± 0.0002	0.0720 ± 0.0000	4.019 ± 0.286
Dietlat desnatado (L30)	6.8179 ± 0.7231	0.2441 ± 0.0929	0.0330 ± 0.0000	2.118 ± 0.114
Parmalat Zymil (L31)	5.7937 ± 1.7356	0.0927 ± 0.0163	0.0171 ± 0.0023	2.119 ± 0.072
Batavo sensy (L32)	4.0326 ± 0.1260	0.0766 ± 0.0251	0.0573 ± 0.0300	1.926 ± 0.065
Batavo cálcio light (L33)	4.0046 ± 0.2861	0.1211 ± 0.0736	0.0173 ± 0.0018	1.541 ± 0.076
Batavo ferro (L34)	4.3945 ± 0.2825	---------	0.0083 ± 0.0043	1.092 ± 0.083
Carrefour semidesnatado (L35)	3.0400 ± 0.1791	0.0809 ± 0.0313	---------	1.086 ± 0.074
Batavo semidesnatado (L36)	3.3878 ± 0.1851	0.1059 ± 0.0254	0.0017 ± 0.0000	1.595 ± 0.084
Piracanjuba desnatado (L37)	2.8128 ± 0.7274	0.1111 ± 0.0281	0.0071 ± 0.0000	0.919 ± 0.086
Compleite integral (L38)	1.8559 ± 0.3373	0.0178 ± 0.0004	0.0571 ± 0.0011	1.020 ± 0.082
Compleite desnatado (L39)	3.1868 ± 0.4768	0.0981 ± 0.0210	0.0058 ± 0.0000	2.826 ± 0.175
Batavo integral (L40)	2.6017 ± 0.1867	0.0630 ± 0.0085	---------	1.383 ± 0.084
Leitbom desnatado (L41)	1.8538 ± 0.2670	0.1237 ± 0.0056	---------	1.682 ± 0.092
Parmalat semidesnatado (L42)	2.6025 ± 0.1138	0.1050 ± 0.0075	0.0186 ± 0.0000	1.449 ± 0.106
Itambé semidesnatado (L43)	3.4107 ± 0.3615	0.0569 ± 0.0055	0.0412 ± 0.0000	1.105 ± 0.091
Italac light (L44)	1.6272 ± 0.8564	---------	0.0060 ± 0.0024	1.145 ± 0.028
Carrefour integra (L45)	2.1363 ± 0.0491	---------	0.0060 ± 0.0044	0.660 ± 0.056
Piracanjuba integral (L46)	2.8251 ± 0.1588	0.1089 ± 0.0725	0.0157 ± 0.0069	0.998 ± 0.028
Marajoara desnatado (L47)	1.7179 ± 0.3872	0.0866 ± 0.0243	0.0321 ± 0.0000	0.443 ± 0.033
Carrefour desnatado (L48)	2.0203 ± 0.1637	0.0752 ± 0.0213	0.0441 ± 0.0000	0.244 ± 0.005
Batavo desnatado (L49)	3.2939 ± 0.3178	0.0879 ± 0.0346	0.0129 ± 0.0044	0.631 ± 0.012
Parmalat ferro (L50)	11.2455 ± 0.9657	0.0231 ± 0.0048	0.0141 ± 0.0033	0.969 ± 0.058
Leitbom integral (L51)	1.9374 ± 0.6540	0.1034 ± 0.0320	0.0771 ± 0.0052	0.610 ± 0,035
Manacá light (L52)	6.1996 ± 0.5509	---------	0.0091 ± 0.0016	0.503 ± 0.027
Paulista semidesnatado (L53)	10.4534 ± 0.0050	---------	0.0040 ± 0.0004	0.405 ± 0.036
Extra integral (L54)	2.5893 ± 0.3633	0.1271 ± 0.0714	0.0121 ± 0.0049	0.712 ± 0.038
Escolha econômica desn. (L55)	2.8792 ± 0.9558	---------	---------	0.281 ± 0.025

a Mean values from three independent determinations.

**Table 4:** Principal component analysis.

Component	Variance (%)	Cumulative variance (%)

1	47.84	47.84
2	23.12	70.96
3	19.02	89.97
4	10.03	100.00
